# Negatively-Doped Single-Walled Carbon Nanotubes Decorated with Carbon Dots for Highly Selective NO_2_ Detection

**DOI:** 10.3390/nano10122509

**Published:** 2020-12-14

**Authors:** Namsoo Lim, Jae-Sung Lee, Young Tae Byun

**Affiliations:** 1 Sensor System Research Center, Korea Institute of Science and Technology (KIST), Seoul 02792, Korea; namsoo@kist.re.kr; 2 Advanced Semiconductor Research Center, Gumi Electronics & Information Technology Research Institute (GERI), Gumi 39253, Korea; jslee1245@geri.re.kr

**Keywords:** Carbon dots, single-walled carbon nanotubes, nitrogen dioxide, gas sensor

## Abstract

In this study, we demonstrated a highly selective chemiresistive-type NO_2_ gas sensor using facilely prepared carbon dot (CD)-decorated single-walled carbon nanotubes (SWCNTs). The CD-decorated SWCNT suspension was characterized using transmission electron microscopy (TEM), X-ray diffraction (XRD), and UV-visible spectroscopy, and then spread onto an SiO_2_/Si substrate by a simple and cost-effective spray-printing method. Interestingly, the resistance of our sensor increased upon exposure to NO_2_ gas, which was contrary to findings previously reported for SWCNT-based NO_2_ gas sensors. This is because SWCNTs are strongly doped by the electron-rich CDs to change the polarity from *p*-type to *n*-type. In addition, the CDs to SWCNTs ratio in the active suspension was critical in determining the response values of gas sensors; here, the 2:1 device showed the highest value of 42.0% in a sensing test using 4.5 ppm NO_2_ gas. Furthermore, the sensor selectively responded to NO_2_ gas (response ~15%), and to other gases very faintly (NO, response ~1%) or not at all (CO, C_6_H_6_, and C_7_H_8_). We propose a reasonable mechanism of the CD-decorated SWCNT-based sensor for NO_2_ sensing, and expect that our results can be combined with those of other researches to improve various device performances, as well as for NO_2_ sensor applications.

## 1. Introduction

As hazardous gases, nitrogen oxides (typically, NO and NO_2_) are mainly generated by combustion processes of fossil fuels, such as vehicle exhausts, power plants, and various industrial processes, and are the main causes of acid rain and photochemical smog, having a significant influence on air, water, and soil pollution [1,2,3]. Furthermore, the gases can cause serious problems to human organisms, irritating eyes, causing dizziness, and chronically weakening the respiratory system. Generally, NO gas is highly reactive due to its radical structure and is oxidized in air into toxic reddish-brown NO_2_ gas with a biting odor, which can lead to death at a concentration above the immediate danger to life and health (IDLH) value of 20 ppm [4]. Therefore, developing a high-performance NO_2_ gas sensor is very important in the fields of human respiratory health and environmental pollution.

For NO_2_ gas detection, electrochemical-, semiconductor (SC, or chemiresistive)-, and infrared (IR) absorption-type sensors are generally used. Among them, the SC-type sensors have been widely studied because of their advantages, such as their rapid detection, wide sensing range, low power consumption, low cost, etc. [5] SC-type gas sensors detect target gases through redox reactions between gas molecules and SC channels. As channel platform materials, metal oxides, conducting polymers (CPs), and carbon-based nanomaterials are typically utilized [5]. The metal oxide-based gas sensors (e.g., ZnO [6,7,8,9], SnO_2_ [10], In_2_O_3_ [11], NiO [12], and WO_3_ [13]) have advantages such as a high responsivity, excellent thermal stability, low cost, etc. On the other hand, they generally require a heating process (>300 °C) to activate the sensing materials, thus increasing the volume of sensor systems and power consumption during operation [5]. CP-based gas sensors (e.g., polyaniline [14]) exhibit moderate response values and rapid detection, but they also have drawbacks, such as long-term instability and irreversibility originating from organic properties, which restrict their practical applications [14,15].

Gas sensors using carbon-based nanomaterials (e.g., single-walled carbon nanotubes (SWCNTs) [5,15,16,17,18,19,20], multi-walled carbon nanotubes (MWCNTs) [3,21], graphene [22,23], graphene oxide (GO) [24], and reduced GO (R-GO)) show desirable properties, such as a high response, detectability of low concentrations, low temperature operations, etc., making them highly attractive as platform materials. However, they still have limitations, such as a low selectivity and long response and recovery times compared to metal oxide- or CP-based sensors [25,26,27,28]. Regarding these issues, there have been some results improved by surface treatments [16,29,30], functionalization [19,31,32,33], the use of core-shell structures [34,35], specially designed hetero-structures [36,37], etc.

In this paper, we propose carbon dot (CD)-decorated SWCNTs as an NO_2_ gas sensing material with a reasonable sensing mechanism. Synthesized CDs and the CD-decorated SWCNTs were characterized by transmission electron microscope (TEM) images and X-ray diffraction (XRD)- and UV-visible spectra. Interestingly, our experimental results show that the sensing platform (i.e., CD-decorated SWCNTs) behaves as an *n*-type material, which is opposite to what has been reported for SWCNT-based gas sensors [3,15]. Moreover, the CDs to SWCNTs ratio in active suspension is a critical factor determining the response value. The NO_2_ gas sensor fabricated with the 2:1 (CDs:SWCNTs) suspension exhibited the highest response of ~42% to 4.5 ppm NO_2_, and responded to a low concentration of 100 ppb (with the response of ~3.3%). More desirably, the sensor insignificantly responded to nitric oxide (NO), and did not respond to carbon monoxide (CO), benzene (C_6_H_6_), and toluene (C_7_H_8_), meaning that it has a high selectivity to NO_2_ gas. Our new proposed mechanism of NO_2_ gas detection can provide researchers of sensor materials and/or devices with a promising solution to further enhance their sensor performances.

## 2. Materials and Methods

### 2.1. Synthesis of Carbon Dots (CDs)

The carbon dots (CDs) were synthesized via a precursor pyrolysis method [38,39]. 1-octadecene (15 mL) and oleylamine (2 mL) were blended in a three-neck flask (50 mL), and then degassed under nitrogen (N_2_) purge for 30 min. Sequentially, the temperature was elevated to 200 °C, and citric acid (1 g, precursor) was added into the flask with mild stirring. After 20 min, the reactant solution was cooled down to room temperature, and ethanol (20 mL) was added for the precipitation of CDs. The solution was centrifuged at 4000 RPM for 10 min, and the precipitated CDs were re-dispersed in 1,2-dichlorobenzene (C_6_H_4_Cl_2_, Sigma-Aldrich, St. Louis, MO, USA) by 0.02 mg/mL.

### 2.2. Preparation of Carbon Dot (CD)-Decorated SWCNT Suspensions

Purchased SWCNTs (diameter: 1.2–1.7 nm, length: 0.1–4 μm, purity: >99%, Nanointegris Technologies, Boisbriand, Quebec, Canada) were used without further purification. A total of 1 mg of the SWCNTs was uniformly dispersed in 50 mL of 1,2-dichlorobenzene by sonicating for 4 h (here, the concentration value was chosen in previously reported SWCNT-based sensors [15,17,18]). For preparing CD-decorated SWCNT suspensions, the CD suspension (0.02 mg/mL, prepared in experimental Section 2.1) was injected into 0.02 mg/mL of SWCNT suspension with the volume ratios of 1:1, 2:1, and 3:1, respectively.

### 2.3. Fabrication of CD-Decorated SWCNT-Based Gas Sensors

**Figure 1:**
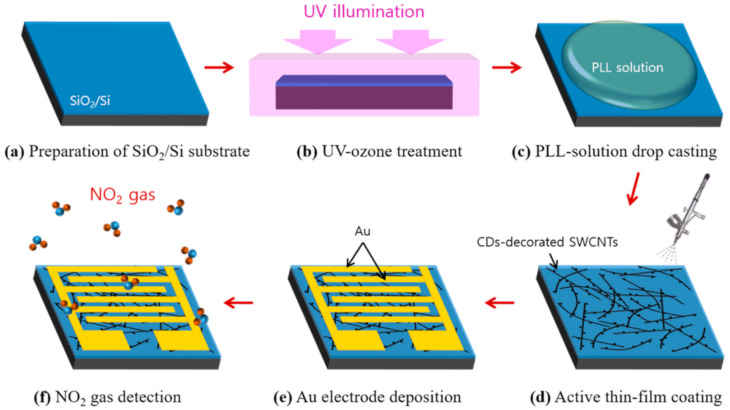
Schematic fabrication process of the chemiresistive-type gas sensor. (**a**) Preparation of the SiO_2_/Si substrate by sonicating it in acetone, methanol, and deionized (DI) water for 10 min each. (**b**) UV-ozone treatment to eliminate organic residues on the SiO_2_/Si substrate. (**c**) poly-l-lysine (PLL)-solution drop casting for the successive carbon dot (CD)-decorated single-walled carbon nanotubes (SWCNT) thin-film formation. (**d**) Active thin-film coating by a spray-printing method. (**e**) Au electrode deposition by a sputtering method. (**f**) Measurement of the NO_2_ gas response in a custom-built gas sensing system.

Figure 1 shows the fabrication schematics of the CD-decorated SWCNT-based gas sensor. An SiO_2_/Si substrate was cleaned by sonicating it in acetone, methanol, and deionized (DI) water for 15 min each, and then exposing it to an ultraviolet (UV)-ozone atmosphere for 20 min to eliminate residual contaminants and make the surface hydrophilic. Sequentially, the SiO_2_ surface was pre-treated with poly-l-lysine (PLL) solution for 20 min to form a homogeneous SWCNT layer [40]. In total, 4 mL of the CD-decorated SWCNT suspension was sprayed onto the SiO_2_/Si substrate using a spray gun with a 0.18 mm-nozzle. The SWCNT network was successfully adsorbed onto the substrate by placing it on a hot plate at 180 °C for 30 min. Finally, interdigitated 200 nm-thick Au electrodes were deposited using a shadow mask with a 150 μm gap.

### 2.4. Sensing Measurements

The gas sensing performance was measured in a custom-built system consisting of gas bombes, mass flow controllers (MFCs), a gas mixer, a gas chamber, etc., as described in Figure S1. Five target gases (NO_2_, CO, NO, C_6_H_6_, and C_7_H_8_) were tested, and each gas was diluted with a carrier gas (N_2_) using accurate MFCs. In all measurements, the total flow rate was 300 sccm at room temperature, and the bias voltage was 1 V. To connect the Keithley 2400 source meter (Keithley Instrument, Cleveland, OH, USA) to a computer, a GPIB-to-USB converter was used, and the LabView software (National Instruments, Austin, TX, USA) was then utilized for data acquisition.

## 3. Results and Discussion

**Figure 2:**
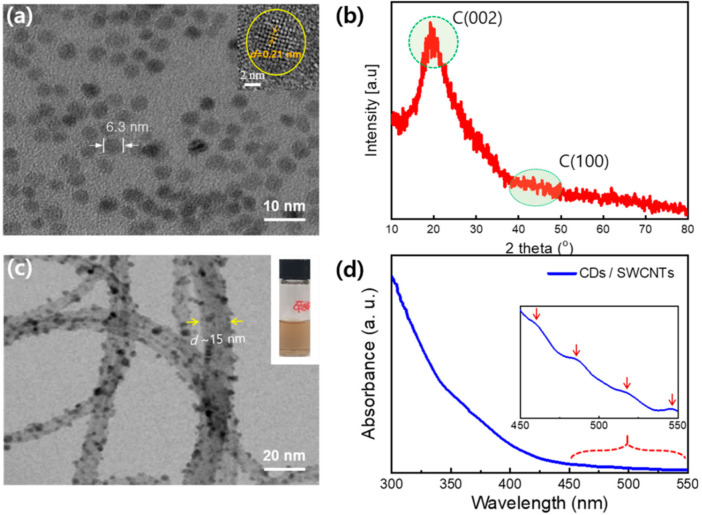
Characterization of carbon dots (CDs) and CD-decorated SWCNTs: (**a**) A transmission electron microscope (TEM) image (inset shows the lattice fringe of a CD); (**b**) a wide-angle X-ray diffraction (XRD) pattern of the synthesized CDs; (**c**) a magnified TEM image of CD-decorated SWCNTs; and (**d**) UV-vis absorbance spectra of the CD-decorated SWCNT suspension (inset shows the magnified spectrum of the 450–550 nm range).

The synthesized carbon dots (CDs) were characterized by TEM analysis. Figure 2a shows that spherical CDs with a uniform diameter of ~6.3 nm were synthesized by the precursor pyrolysis method. The high resolution TEM image (inset) shows the lattice fringes of a CD, whose *d*-spacing value is 0.21 nm, indicating the (100) lattice structure [1]. The CDs were further characterized by a wide-angle X-ray diffraction (XRD) pattern (Figure 2b). Two broad peaks (including a faint and broad peak at around 2*θ* = 42°) demonstrate the existence of numerous disordered CDs containing *C*(002) and *C*(100) lattice structures [41].

Three kinds of CD-decorated SWCNT suspensions were prepared with volume ratios of 1:1, 2:1, and 3:1, respectively, and then characterized using TEM and UV-visible spectroscopy. Figure 2c is a highly magnified TEM image of the (1:1) CD-decorated SWCNTs. Here, strands of SWCNTs decorated with lots of CDs are clearly seen, verifying the successful decoration of SWCNTs with CDs. Because there is no chemical interaction between CDs and SWCNTs, the CDs are weakly bound to SWCNT walls by van der Waals forces [1]. Considering that the SWCNTs with diameters of 1.2–1.7 nm were initially used, the diameter of ~15 nm in the TEM image was obtained as a bundle of SWCNTs, but not an individual SWCNT (TEM images of the SWCNTs before and after CD decoration are compared in Figure S2). The inset shows a photograph of the CD-decorated SWCNT suspension with a brownish color, which demonstrates its homogeneous dispersion.

Figure 2d shows the UV-visible absorbance spectrum of the CD-decorated SWCNT suspension. The spectrum looks similar to that of a pure CD suspension, whose absorbance continuously increases down to 300 nm [41]. However, a series of tiny peaks were found in the wavelength region of 450~550 nm (see the inset). The small peaks indicated by red arrows are only observed in the SWCNT suspension, and are not found in the pure CD suspension. This is more evidence for CD-decorated SWCNTs (see Figure S3 for more information).

**Figure 3:**
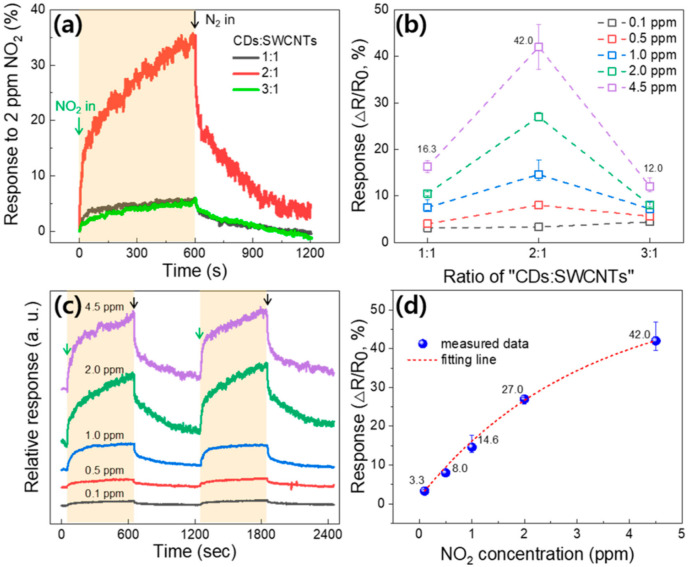
(**a**) Time-resolved response curves of gas sensors with CDs to SWCNTs ratios of 1:1, 2:1, and 3:1, respectively. (**b**) Response variations depending on the CDs to SWCNTs ratio. (**c**) Variations of the time-resolved response curves at different NO_2_ concentrations. (**d**) Response variation of the 2:1 device as a function of the NO_2_ concentration.

Three kinds of gas sensors were fabricated using the different CD-decorated SWCNT suspensions (1:1, 2:1, and 3:1), and the responses to the nitrogen dioxide (NO_2_) gas were measured in the custom-built gas sensing system (see Figure S1 for fabrication details). Figure 3a shows the time-resolved response curves of three sensors measured at 2 ppm NO_2_ (here, the gas response (%) is defined as $\left(\Delta R/R_{0}\right)\times 100$). During the measurement, the NO_2_ gas was introduced into the chamber consistently with a total flow rate of 300 sccm by using a mass flow controller (MFC) for 600 s (on state), and the inert nitrogen (N_2_) gas was then introduced identically (off state). All the measurements were performed at room temperature (~25 °C). Here, since the CD-decorated SWCNT-based sensors do not show any saturations in both the on and off states, the response and recovery times are defined as the times required to reach and recover 90% of the sensor’s maximum and minimum resistances, respectively [15,17,21]. According to the analyzed data, the average response and recovery times are ~381 and ~294 s, respectively, and there is no noticeable dependence of the response and recovery time on the CDs to SWCNTs ratio or NO_2_ concentration (see Figure S4 for more information).

In general, the resistance of an SWCNT-based chemiresistive-type gas sensor decreases when exposed to the oxidizing NO_2_ gas. This is because SWCNTs display a *p*-type nature in air due to the doping effects of H_2_O and O_2_ [42], and electron-withdrawing NO_2_ molecules capture electrons from SWCNTs, making more holes in the *p*-type channel, as described in Equation (1) [3,15].

(1) $${\mathrm{NO}}_{2}\to {\;\mathrm{NO}}_{2}{}^{-}+{\;h}^{+}$$

Interestingly, in our results, the sensor resistance increased when exposed to NO_2_, as shown in Figure 3a. This phenomenon can be understood with the *n*-doping effect by the electron-rich CDs [39,43]. The decrease in electron density upon exposure to NO_2_ gas can increase the resistance of CD-decorated SWCNT sensors. Moreover, the results show that the CDs to SWCNTs ratio is critical in determining the response values. The sensor’s response values extracted from their time-resolved response curves are summarized in Figure 3b. When the CDs to SWCNTs ratio is 2:1, the sensor shows the highest response (R_average_) value of ~42.0% to NO_2_ gas at 4.5 ppm. From the results, we can reasonably expect that there exists an optimized CDs to SWCNTs ratio for NO_2_ gas molecule adsorption, which means the optimum doping state of the SWCNTs for NO_2_ detection. In this experiment, we controlled the NO_2_ concentration in target gas at 0.1, 0.5, 1.0, 2.0, and 4.5 ppm, and then measured the time-resolved response curves in each case. The results show that at every concentration of NO_2_ gas (except the 0.1 ppm case, in which the response difference is not clear), the 2:1 sensor exhibited the highest response values.

Figure 3c shows relative comparisons of the time-resolved response curves measured at different NO_2_ concentrations (hereafter, the 2:1 sensor was used for analysis). Shaded regions indicate the area where the NO_2_ gas was introduced. At the low concentrations of 0.1 and 0.5 ppm, the difference in their relative response values is inconspicuous. However, as the NO_2_ concentration increases, the corresponding response value markedly increases. Figure 3d shows plotted data of the response values as a function of the NO_2_ concentration. At each concentration, 5-cycle response curves were measured and their response values were averaged. The red-dotted curve is a fitting line with the formula $y=A_{1}e^{\left(-\frac{x}{t_{1}}\right)}+A_{2}e^{\left(-\frac{x}{t_{2}}\right)}+\;y_{0}$ (here, the fitting line coincides well with the actual data, with the coefficient of determination (COD, R^2^) value of 0.9969). When the NO_2_ concentration increases from 0.1 to 2.0 ppm, the gas response rapidly increases from 3.3% to 27.0%. In this concentration range of NO_2_ gas, surface occupation by the NO_2_ gas molecules is accelerated in a relatively short time, markedly increasing the surface reaction [3,44]. However, in the range of 2.0~4.5 ppm, the surface reaction becomes gradual, and the response increases slightly to 42.0%. Through the results, it can be predicted that the active surface coverage is gradually saturated by NO_2_ molecules at concentrations above 2.0 ppm, and the number of vacant adsorption sites thus decreases. From the curve, the sensor’s limit of detection (LOD) was theoretically calculated to be 18 ppb [1,45] (see Figure S5 for more information).

**Figure 4:**
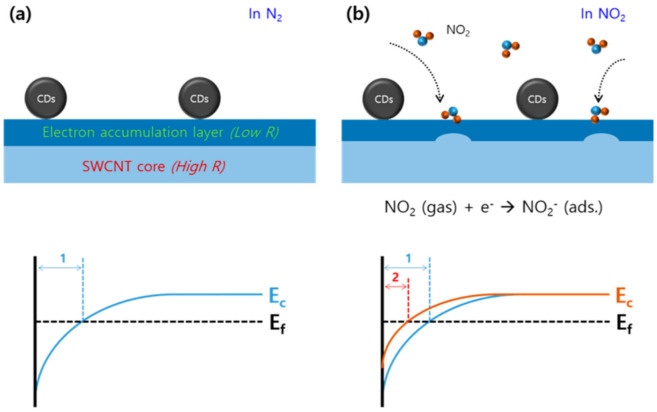
Schematic illustration of the expected NO_2_ gas-sensing mechanism of the CD-decorated SWCNTs: (**a**) In an inert atmosphere, and (**b**) upon exposure to NO_2_. The band diagrams indicate the schematic electron distributions of the corresponding structures (bending parts (1 and 2) correspond to the electron accumulation layers in each case).

Figure 4 describes an expected NO_2_ gas sensing mechanism of the CD-decorated SWCNT-based sensor. As discussed above, the CD-decorated SWCNTs behave as *n*-type material because the SWCNTs are heavily doped by the electron-rich CDs. Considering that the SWCNT is a hollow structure, the carriers are mainly present near the wall, which increases the carrier concentration (Figure 4a). This state can be expressed as band-bending, as shown in the diagram below. The electron accumulation layer corresponds to the bending area (1). In the presence of NO_2_ gas, oxidative NO_2_ molecules are adsorbed on the SWCNT surface, resulting in electron transfer from the SWCNT surface to the NO_2_ molecules. Therefore, the concentration of electrons (i.e., main carriers) is decreased, as schematically shown in Figure 4b. Meanwhile, the adsorption of NO_2_ gas molecules on the surface of SWCNTs causes upward band-bending in the energy band (1 → 2), which results in an increase of the electrical resistance.

**Figure 5:**
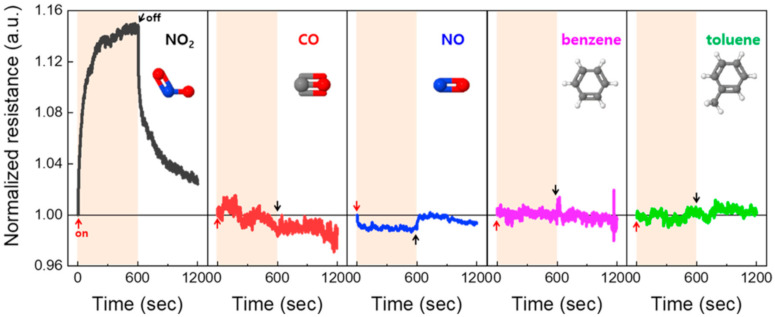
Selectivity test of the CD-decorated SWCNT-based gas sensor (CDs:SWCNTs = 2:1); normalized resistance (=*R*/*R*_0_) vs. time curves of gas sensors for five gas species (1 ppm of NO_2_, CO, NO, C_6_H_6_, and C_7_H_8_). The measurements were carried out utilizing the custom-built sensing system at room temperature. The inset images indicate the corresponding gas molecular structures.

Finally, we measured the sensing performances of the CD-decorated SWCNT-based gas sensor for five different gases (NO_2_, CO, NO, C_6_H_6_, and C_7_H_8_) to evaluate the sensor’s selectivity. Here, each target gas was diluted by using N_2_ gas to obtain a concentration of 1.0 ppm, and the resistance change was then measured by the same method. Figure 5 shows the time-resolved response curves of the sensor for the above-mentioned gas species (here, the normalized resistance value (*y*-axis) is defined as *R/R_0_*, and each inset image indicates the visualized molecular structure of the corresponding gas). In the case of NO_2_ gas, the resistance increases immediately upon exposure to the gas, and also rapidly decreases when N_2_ gas is introduced, with the maximum value of ~1.15 (converted response value is ~15%). The large resistance variation can be understood with the electrophilic nature of NO_2_ molecules that can easily interact with the *n*-type channel, as discussed above. On the other hand, there is no noticeable resistance change for other gases (CO, C_6_H_6_, and C_7_H_8_), except for the NO gas (~1%). The slight decrease in the resistance upon exposure to NO gas is probably due to the electron-donating effect of the NO molecules [46]. When the NO molecules are adsorbed on the surface of CD-decorated SWCNTs, the NO molecules are oxidized to give electrons to the *n*-type channel. As a result, the sensor’s resistance decreases. Nevertheless, because the response value to NO gas is negligible (~1%) compared to that of NO_2_ gas (~15%), we can conclude that the CD-decorated SWCNT-based sensor can detect NO_2_ gas with a high selectivity.

## 4. Conclusions

In this paper, we have suggested the possibility of employing CD-decorated SWCNTs for highly selective NO_2_ detection at room temperature. The facilely prepared CD-decorated SWCNT suspension was spray-coated on an SiO_2_/Si substrate, and the interdigitated Au electrodes were then sputtered to complete the sensor. The resistance of the sensor increased when exposed to NO_2_ gas, which was opposite to the results of previously reported SWCNT-based NO_2_ sensors. This is because the SWCNTs were heavily *n*-doped by the synthesized CDs. In addition, the response values of the sensors were significantly changed, depending on the CDs to SWCNTs ratio in the active suspension, and showed the highest value of 42% at the 2:1 ratio (in results of the sensing test using 4.5 ppm NO_2_). More desirably, the fabricated sensor responded very weakly to NO gas, and did not respond at all to other gases (CO, C_6_H_6_, and C_7_H_8_), revealing its high selectivity toward NO_2_ gas. We expect that the proposed NO_2_ sensing mechanism and the doping phenomenon can be utilized to improve various device performances, as well as highly selective NO_2_ sensor applications.

## Supplementary Materials

The following are available online at https://www.mdpi.com/2079-4991/10/12/2509/s1, Figure S1: Schematic image of the chemiresistive-type gas sensor measurement system, Figure S2: TEM images of SWCNTs; (**a**) before- and (**b**) after the CDs decoration, Figure S3: UV-visible spectra of the synthesized carbon dots (CDs) (**a**) and the purchased single-walled carbon nanotubes (SWCNTs) (**b**). (Each inset shows a photograph of the corresponding suspension.), Figure S4: (**a**) Definition of response- and recovery times in a typical time-resolved response curve. Variations of (**b**) response- and (**c**) recovery times depending on the ratio of CDs to SWCNTs (the measurement was performed at 2.0 ppm of NO_2_ concentration.). Variations of (**d**) response- and (**e**) recovery times depending on the NO_2_ concentrations (the 2:1 device was utilized for this data.), Figure S5: (**a**) Time-resolved response curve of the sensor to 0.1 ppm NO_2_. (**b**) Response to NO_2_ concentration curve and its fitting.

## References

[1] Cheng M., Wu Z., Liu G., Zhao L., Gao Y., Li S., Zhang B., Yan X., Lu G. (2020). Carbon dots decorated hierarchical litch-like In_2_O_3_ nanospheres for highly sensitive and selective NO_2_ detection. Sens. Actuators B.

[2] Wang R., Li G., Dong Y., Chi Y., Chen G. (2013). Carbon quantum dot-functionalized aerogels for NO_2_ gas sensing. Anal. Chem..

[3] Sayago I., Santos H., Horrillo M.C., Aleixandre M., Fernandez M.J., Terrado E., Tacchini I., Aroz R., Maser W.K., Benito A.M. (2008). Carbon nanotube networks as gas sensors for NO_2_ detection. Talanta.

[4] Mane A.A., Moholkar A.V. (2018). Palladium (Pd) sensitized molybdenum trioxide (MoO_3_) nanobelts for nitrogen dioxide (NO_2_) gas detection. Solid-State Electron..

[5] Jeon J., Kang B., Byun Y.T., Ha T. (2019). High-performance gas sensors based on single-walled carbon nanotube random networks for the detection of nitric oxide down to the ppb-Level. Nanoscale.

[6] Pan X., Liu X., Bermak A., Fan Z. (2013). Self-gating effect induced large performance improvement of ZnO nanocomb gas sensors. ACS Nano.

[7] Sun G., Lee J.K., Choi S., Lee W.I., Kim H.W., Lee C. (2017). Selective oxidizing gas sensing and dominant sensing mechanism of n-CAO-decorated n-ZnO nanorod sensors. ACS Appl. Mater. Interfaces.

[8] Shishiyanu S.T., Shishiyanu T.S., Lupan O.I. (2005). Sensing characteristics of tin-doped ZnO thin films as NO_2_ gas sensor. Sens. Actuators B.

[9] Kumar R., Al-Dossary O., Kumar G., Umar A. (2015). Zinc oxide nanostructures for NO_2_ gas-sensor applications: A review. Nano-Micro Lett..

[10] Li Y.-X., Guo Z., Su Y., Jin X.-B., Tang X.-H., Huang J.-R., Huang X.-J., Li M.-Q., Liu J.-H. (2017). Hierarchical morphology-dependent gas-sensing performances of three-dimensional SnO_2_ nanostructures. ACS Sens..

[11] Wang X., Su J., Chen H., Li G.-D., Shi Z., Zou H., Zou X. (2017). Ultrathin In_2_O_3_ nanosheets with uniform mesopores for highly sensitive nitric oxide detection. ACS Appl. Mater. Interfaces.

[12] Zhang J., Zeng D., Zhu Q., Wu J., Xu K., Liao T., Zhang G., Xie C. (2015). Effect of grain-boundaries in NIO nanosheet layers room-temperature sensing mechanism under NO_2_. J. Phys. Chem. C.

[13] Cai Z.-X., Li H.-Y., Ding J.-C., Guo X. (2017). Hierarchical flowerlike WO_3_ nanostructures assembled by porous nanoflakes for enhanced NO gas sensing. Sens. Actuators B.

[14] Geng L., Zhao Y., Huang X., Wang S., Zhang S., Wu S. (2007). Characterization and gas sensitivity study of polyaniline/SnO_2_ hybrid material prepared by hydrothermal route. Sens. Actuators B.

[15] Choi S.-W., Kim J., Byun Y.T. (2017). Highly sensitive and selective NO_2_ detection by Pt nanoparticles-decorated single-walled carbon nanotubes and the underlying sensing mechanism. Sens. Actuators B.

[16] Kim J., Choi S.-W., Lee J.-H., Chung Y., Byun Y.T. (2016). Gas sensing properties of defect-induced single-walled carbon nanotubes. Sens. Actuators B.

[17] Choi S.-W., Byun Y.T. (2018). The effect of platinum precursor concentrations on chlorine sensing characteristics of platinum nanoparticles-loaded single walled carbon nanotubes. Appl. Surf. Sci..

[18] Lee D.-J., Choi S.-W., Byun Y.T. (2018). Room temperature monitoring of hydrogen peroxide vapor using platinum nanoparticles-decorated single-walled carbon nanotube networks. Sens. Actuators B.

[19] Choi S.-W., Kim B.-M., Oh S.-H., Byun Y.T. (2017). Selective detection of chlorine at room temperature utilizing single-walled carbon nanotubes functionalized with platinum nanoparticles synthesized via ultraviolet irradiation. Sens. Actuators B.

[20] Lee J.-S., Jeong D.-W., Byun Y.T. (2020). Porphyrin nanofiber/single-walled carbon nanotube nanocomposite-based sensors for monitoring hydrogen peroxide vapor. Sens. Actuators B.

[21] Yaqoob U., Phan D.-T., Uddin A.S.M.I., Chung G.-S. (2015). Highly flexible room temperature NO_2_ sensor based on MWCNTs-WO_3_ nanoparticles hybrid on a PET substrate. Sens. Actuators B.

[22] Hong J., Lee S., Seo J., Pyo S., Kim J., Lee T. (2015). A highly sensitive hydrogen sensor with gas selectivity using a PMMA membrane-coated Pd nanoparticle/single-layer graphene hybrid. Sens. ACS Appl. Mater. Interfaces.

[23] Chung M.G., Kim D.H., Lee H.M., Kim T., Choi J.H., Seo D., Yoo J.-B., Hong S.-H., Kang T.-J., Kim Y.H. (2012). Highly sensitive NO_2_ gas sensor based on ozone treated graphene. Sens. Actuators B.

[24] Choi S.-J., Ryu W.-H., Kim S.-J., Cho H.-J., Kim I.-D. (2014). Bi-functional co-sensitization of graphene oxide sheets and Ir nanoparticles on p-Type Co_3_O_4_ nanofibers for selective acetone detection. J. Mater. Chem. B.

[25] Septiani N.L.W., Yuliarto B. (2016). Review-the development of gas sensor based on carbon nanotubes. J. Electrochem. Soc..

[26] Ellis J.E., Star A. (2016). Carbon nanotube based gas sensors toward breath analysis. ChemPlusChem.

[27] Goldoni A., Alijani V., Sangaletti L., D’Arsie L. (2018). Advanced promising routes of carbon/metal oxides hybrid in sensors: A review. Electrochim. Acta.

[28] Baptista F.R., Belhout S.A., Giordani S., Quinn S.J. (2015). Recent develpments in carbon nanomaterial sensors. Chem. Soc. Rev..

[29] Zhang X., Yang B., Wang X., Luo C. (2012). Effect of plasma treatment on multi-walled carbon nanotubes for the detection of H_2_S and SO_2_. Sensors.

[30] Zhao W., Fam D.W.H., Yin Z., Sun T., Tan H.T., Liu W., Tok A.I.Y., Boey Y.C.F., Zhang H., Hng H.H. (2012). A carbon monoxide gas sensor using oxygen plasma modified carbon nanotubes. Nanotechnology.

[31] Cho B., Yoon J., Hahm M.G., Kim D.-H., Kim A.R., Kahng Y.H., Park S.-W., Lee Y.-J., Park S.-G., Kwon J.-D. (2014). Graphene-based gas sensor: Metal decoration effect and application to a flexible device. J. Mater. Chem. C.

[32] Evans G.P., Buckley D.J., Skipper N.T., Parkin I.P. (2014). Single-walled carbon nanotube composite inks for printed gas sensors: Enhanced detection of NO_2_, NH_3_, EtOH and acetone. RSC Adv..

[33] Choi S.-W., Kim J., Lee J.-H., Byun Y.T. (2016). Remarkable improvement of CO-sensing performances in single-walled carbon nanotubes due to modification of the conducting channel by functionalization of Au nanoparticles. Sens. Actuators B.

[34] Marichy C., Russo P.A., Latino M., Tessonnier J.-P., Willinger M.-G., Donato N., Neri G., Pinna N. (2013). Tin dioxide-carbon heterostructures applied to gas sensing: Structure-dependent properties and general sensing mechanism. J. Phys. Chem. C.

[35] Tian X., Wang Q., Chen X., Yang W., Wu Z., Xu X., Jiang M., Zhou Z. (2014). Enhanced performance of core-shell structured polyaniline at helical carbon nanotube hybrids for ammonia gas sensor. Appl. Phys. Lett..

[36] Liu S., Wang Z., Zhang Y., Zhang C., Zhang T. (2015). High performance room temperature NO_2_ sensors based on reduced graphene oxide-multiwalled carbon nanotubes-tin oxide nanoparticles hybrids. Sens. Actuators B.

[37] Tung T.T., Pham-Huu C., Janowska I., Kim T.Y., Castro M., Feller J.-F. (2015). Hybrid films of graphene and carbon nanotubes for high performance chemical and temperature sensing applications. Small.

[38] Wang S., Chen Z.-G., Cole I., Li Q. (2015). Structural evolution of graphene quantum dots during thermal decomposition of citric acid and the corresponding photoluminescence. Carbon.

[39] Song Y., Zhu S., Zhang S., Fu Y., Wang L., Zhao X., Yang B. (2015). Investigation from chemical structure to photoluminescent mechanism: A type of carbon dots from the pyrolysis of citric acid and an amine. J. Mater. Chem. C.

[40] Zhang Y., Li J., Shen Y., Wang M., Li J. (2004). Poly-l-lysine functionalization of single-walled carbon nanotubes. J. Phys. Chem. B.

[41] Edison T.N.J.I., Atchundan R., Sethuraman M.G., Shim J.-J., Lee Y.R. (2016). Microwave assisted green synthesis of fluorescent N-doped carbon dots: Cytotoxicity and bio-imaging applications. J. Photochem. Photobiol. B.

[42] Duong D.L., Lee S.M., Lee Y.H. (2012). Origin of unipolarity in carbon nanotubes field effect transistors. J. Mater. Chem..

[43] Zhu W., Song H., Zhang L., Weng Y., Su Y., Lv Y. (2015). Fabrication of fluorescent nitrogen-rich graphene quantum dots by tin(IV) catalytic carbonization of ethanolamine. RSC Adv..

[44] Robinson J.A., Snow E.S., Badescu S.C., Reinecke T.L., Perkins F.K. (2006). Role of defects in single-walled carbon nanotube chemical sensors. Nano Lett..

[45] Kumar D., Chaturvedi P., Saho P., Jha P., Chouksey A., Lal M., Rawat J.S.B.S., Tandon R.P., Chaudhury P.K. (2017). Effect of single wall carbon nanotube networks on gas sensor response and detection limit. Sens. Actuators B.

[46] Hoffmann M.W.G., Prades J.D., Mayrhofer L., Hernandez-Ramirez F., Jarvi T.T., Moseler M., Waag A., Shen H. (2014). Highly selective SAM-nanowire hybrid NO_2_ sensor: Insight into charge transfer dynamics and alignment of frontier molecular orbitals. Adv. Funct. Mater..

