# Supplementary material for: Negatively-Doped Single-Walled Carbon Nanotubes Decorated with Carbon Dots for Highly Selective NO2 Detection
Source: Nanomaterials (Basel). 2020 Dec 14;10(12):2509. doi: 10.3390/nano10122509 (PMC7764981; doi:10.3390/nano10122509)
Supplement: Supplementary file 1 [file nanomaterials-10-02509-s001.pdf]

nanomaterials

MDPI

# Supplementary Materials

# Negatively-Doped Single-Walled Carbon Nanotubes Decorated with Carbon Dots for Highly Selective $NO_2$ Detection

**Namsoo Lim [1,†], Jae-Sung Lee [2,†] and Young Tae Byun [1,*]**

1 Sensor System Research Center, Korea Institute of Science and Technology (KIST), Seoul 02792, Korea; namsoo@kist.re.kr
2 Advanced Semiconductor Research Center, Gumi Electronics & Information Technology Research Institute (GERI), Gumi 39253, Korea; jslee1245@geri.re.kr
* Correspondence: byt427@kist.re.kr; Tel.: +82-(0)2-958-5797
† These authors contributed equally to this work.

**S1. Gas sensor measurement system.**

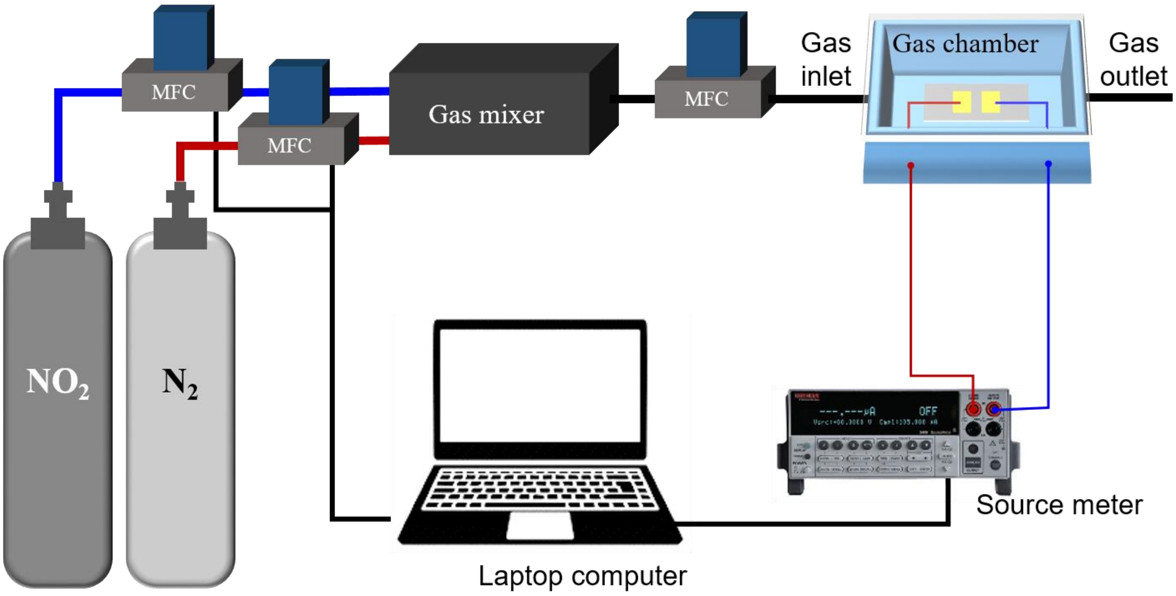


**Figure S1.** Schematic image of the chemiresistive-type gas sensor measurement system.

**S2. Comparison of TEM images; SWCNTs *before*- and *after* the CDs decoration.**

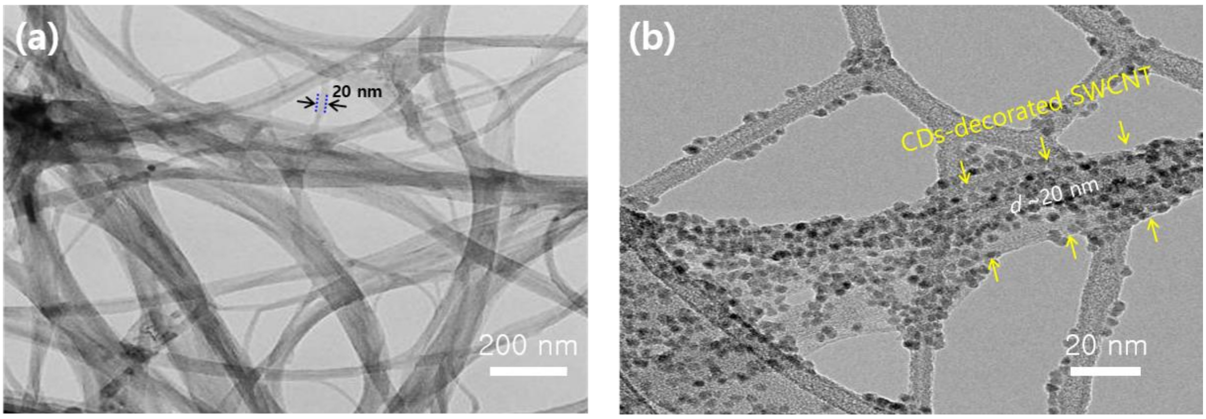


**Figure S2.** TEM images of SWCNTs; **(a)** before- and **(b)** after the CDs decoration.

Purchased SWCNTs (Nanointegris Technologies, Inc., Canada) were utilized as precursors for active material preparation. Before the CDs decoration (Figure S2a), pure SWCNTs with the measured minimum diameter of ~20 nm were found. Because the SWCNTs with diameters of 1.2 ~ 1.7 nm (Sigma-Aldrich, USA) were initially used, these SWCNTs are determined as SWCNT bundles. After decorating with CDs, the CDs-decorated SWCNTs are clearly seen (Figure S2b). The measured diameter of ~20 nm indicates the SWCNTs in this image are also in bundle state.

**S3. Comparison of UV-visible spectra; carbon dots (CDs) vs. SWCNTs.**

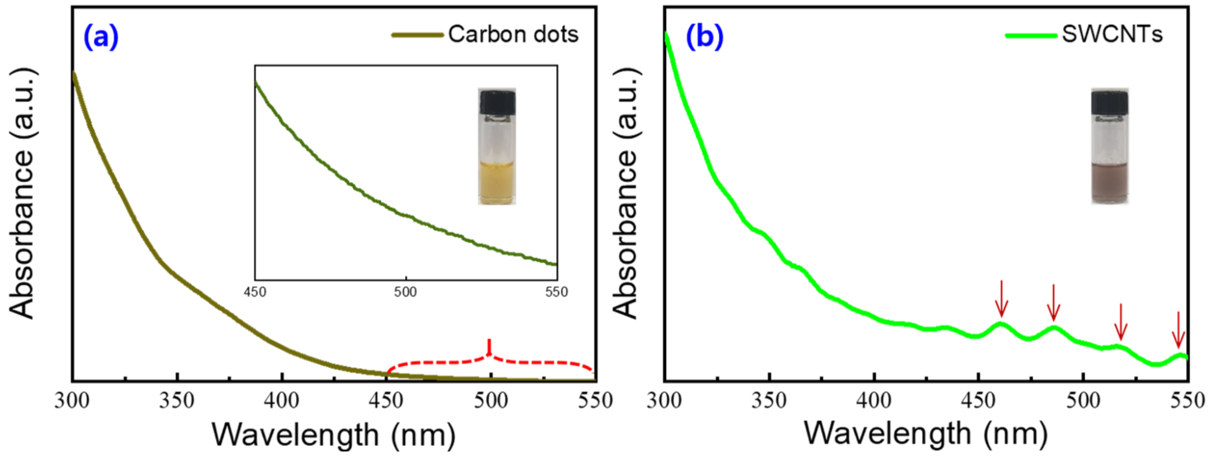


**Figure S3.** UV-visible spectra of the synthesized carbon dots (CDs) (**a**) and the purchased single-walled carbon nanotubes (SWCNTs) (**b**). (Each inset shows a photograph of the corresponding suspension.).

Figure S3a shows a UV-visible spectrum of carbon dots (CDs) suspension dispersed in 1,2-dichlorobenzene. As the wavelength decreases to 300 nm, the absorbance value continuously increases, which is similar to that of CDs-decorated SWCNTs (this is mentioned in the manuscript.). However, being different from the case of CDs-decorated SWCNTs, no small peaks are detected in the spectra of CDs-only suspension (inset is the magnified UV-vis spectrum in the wavelength range of 450~550 nm). Figure S3b shows a UV-visible spectrum of the SWCNTs-only suspension dispersed in 1,2-dichlorobenzene. The small peaks (indicated by four red arrows) are detected more clearly at identical positions. Based on this analysis, the UV-visible spectrum in Figure 2d (in manuscript) is definitely determined as of the CDs-decorated SWCNTs, which demonstrate the successful decoration process.

**S4. Definition of the response- and recovery times, and their variations; dependency on the CDs to SWCNTs ratio and $NO_2$ concentration.**

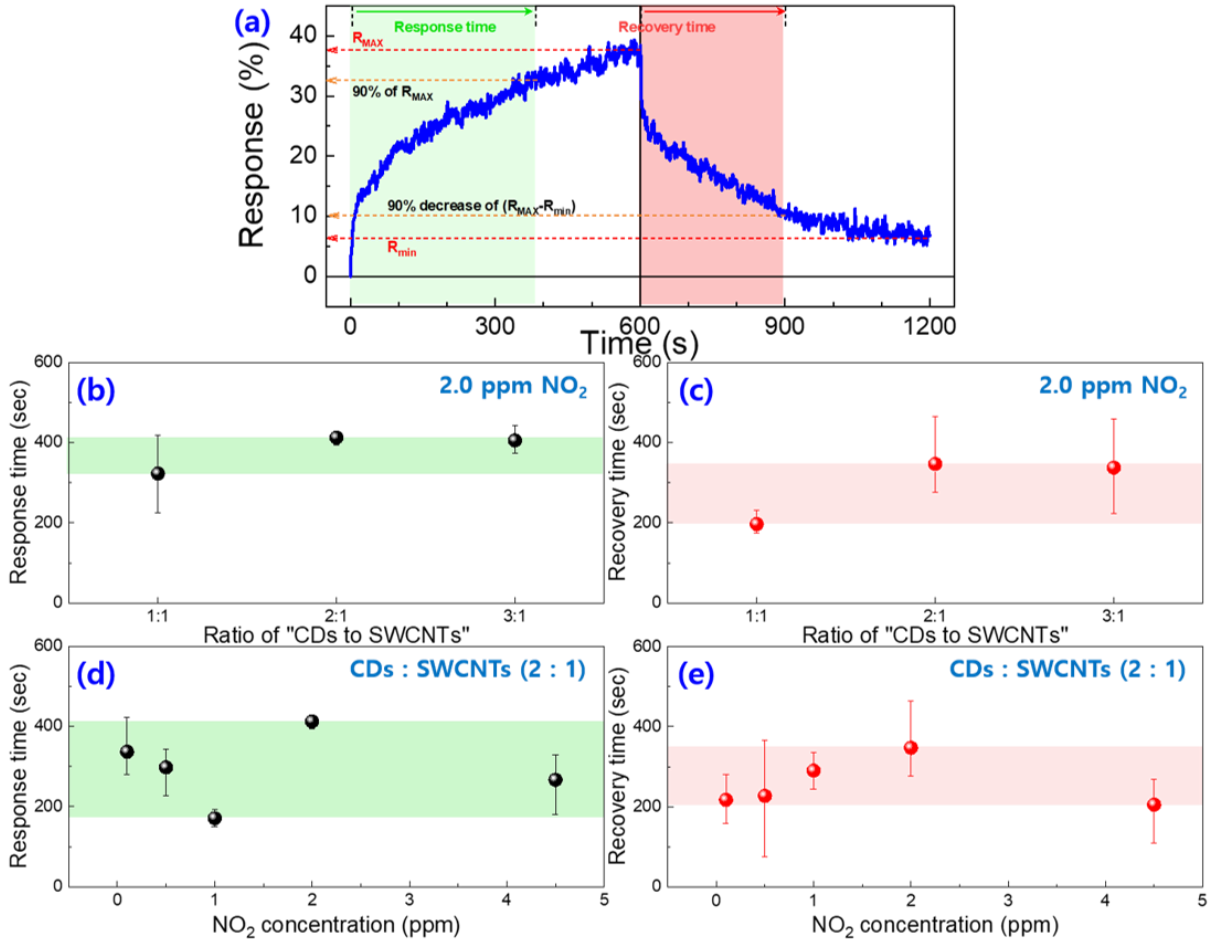


**Figure S4.** (**a**) Definition of response- and recovery times in a typical time-resolved response curve. Variations of (**b**) response- and (**c**) recovery times depending on the ratio of CDs to SWCNTs (the measurement was performed at 2.0 ppm of $NO_2$ concentration.). Variations of (**d**) response- and (**e**) recovery times depending on the $NO_2$ concentrations (the 2:1 device was utilized for this data.).

Figure S4a shows a time-resolved response curve which is obtained from the sensing test using the 2:1 device at 2.0 ppm $NO_2$. For 600 s, the curve does not show any saturations at both the on- and off states. In the case without saturation, the response (or recovery) time is usually defined as the duration time to reach 90% of the $R_{MAX}$ (or $R_{min}$) from turn-on (or turn-off) time [1,2,3]. We have analyzed the variations of the response- and recovery times depending on the CDs to SWCNTs ratio and $NO_2$ concentration. The results show that no noticeable dependency of the response (or recovery) time is seen depending on the CDs to SWCNTs ratio in Figure S4b (or S3c). Also, there is no dependency on the $NO_2$ concentration (Figure S4d (or S4e) shows the summary of the response (or recovery) times.). The average response- and recovery times from all the obtained data are 381 s and 294 s, respectively.

**S5. Theoretical calculation of the sensor's limit of detection (LOD).**

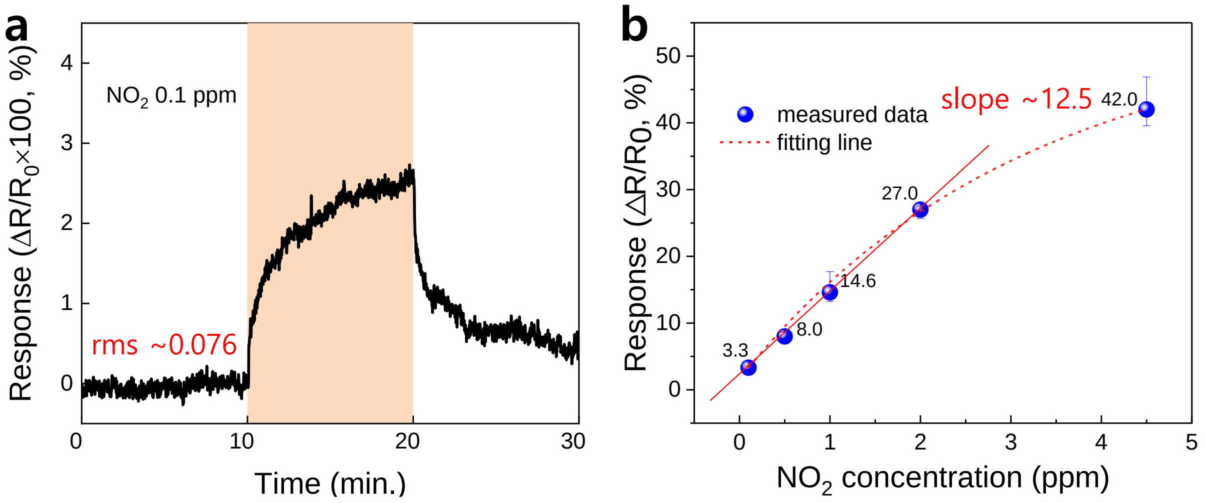


**Figure S5.** (**a**) Time-resolved response curve of the sensor to 0.1 ppm $NO_2$. (**b**) Response to $NO_2$ concentration curve and its fitting.

The limit of detection (LOD) can be calculated by

$$\mathrm{LOD} = \frac{3 \times \mathrm{rms}}{slope},$$

where the slope refers to a line fit to the signal ($\Delta R/R_0$) versus concentration in the sensor's quasi-linear response region (*rms refers to the root-mean-squre noise of the baseline) [4,5].

The "rms" and the slope values are obtained from a time-resolved response curve and the "response-$NO_2$ concentration" curve, respectively (Figure S5a and b). The rms value is calculated using the excel function "STDEV" to 0.076% (a), and the slope is ~12.5%/ppm (b) (the "response-$NO_2$ concentration" curve is linear in 0.1~2 ppm, and the slope is extracted from this range.). Accordingly, the LOD is calculated as,

$$LOD = \frac{3\times 0.076\ [\%]}{12.5\ [\%/ppm]} = 0.01824\ [ppm] \approx 18\ [ppb].$$

## References in Supplementary Materials


1. Choi, S.-W.; Kim, J.; Byun, Y.T. Highly sensitive and selective NO2 detection by Pt nanoparticles-decorated single-walled carbon nanotubes and the underlying sensing mechanism. *Sensors Actuators B: Chem.* **2017**, *238*, 1032–1042.
2. Choi, S.-W.; Byun, Y.T. The effect of platinum precursor concentrations on chlorine sensing characteristics of platinum nanoparticles−loaded single walled carbon nanotubes. *Appl. Surf. Sci.* **2018**, *433*, 480–486.
3. Yaqoob, U.; Phan, D.-T.; Uddin, A.I.; Chung, G.-S. Highly flexible room temperature $NO_2$ sensor based on MWCNTs-$WO_3$ nanoparticles hybrid on a PET substrate. *Sensors Actuators B: Chem.* **2015**, *221*, 760–768.
4. Cheng, M.; Wu, Z.; Liu, G.; Zhao, L.; Gao, Y.; Li, S.; Zhang, B.; Yan, X.; Lu, G. Carbon dots decorated hierarchical litch-like $In_2O_3$ nanospheres for highly sensitive and selective $NO_2$ detection. *Sens. Actuators B: Chem.* **2020**, *304*, 127272.
5. Kumar, D.; Chaturvedi, P.; Saho, P.; Jha, P.; Chouksey, A.; Lal, M.; Rawat, J.; Tandon, R.; Chaudhury, P. Effect of single wall carbon nanotube networks on gas sensor response and detection limit. *Sensors Actuators B: Chem.* **2017**, *240*, 1134–1140.
